# Context-dependent functions of ALKBH5: a mechanistic framework linking cellular stress responses, immune regulation, viral infection, and therapeutic vulnerabilities

**DOI:** 10.3389/fimmu.2026.1716445

**Published:** 2026-05-01

**Authors:** M. Azeem Riaz, Wen-Xuan Li, Yong-Yi Yang, Sehrish Siddique, Chun-Wei Shi, Gui-Lian Yang, Wen-Tao Yang, Chun-Feng Wang

**Affiliations:** College of Veterinary Medicine, Jilin Provincial Engineering Research Center of Animal Probiotics, Jilin Provincial Key Laboratory of Animal Microecology and Healthy Breeding, Engineering Research Center of Microecological Vaccines (Drugs) for Major Animal Diseases, Ministry of Education, Jilin Agricultural University, Changchun, China

**Keywords:** ALKBH5, context-dependent roles, DNA damage response, epitranscriptomic regulation, erasers, N6-methylAdenosine (m6A), T cell biology

## Abstract

*N^6^*-methyladenosine (m^6^A) is a major post-transcriptional RNA modification, and the demethylase ALKBH5 has emerged as a versatile but highly context-dependent regulator of RNA fate. This review integrates current evidence showing that ALKBH5 links epitranscriptomic control to cellular stress adaptation, genome maintenance, immune-cell function, viral infection, and therapeutic response. In DNA damage and cell-cycle regulation, ALKBH5 modulates checkpoint, repair, and apoptotic pathways, thereby influencing genome stability and sensitivity to radiotherapy or chemotherapy. In immune biology, it shapes γδ T-cell development, CD4^+^ T-cell pathogenicity, CD8^+^ T-cell infiltration, and tumor-immune crosstalk. In host-pathogen interactions, ALKBH5 can either enhance antiviral defense or promote viral persistence and latency, including HIV-1 reactivation, depending on the regulated transcript network. We propose that the biological output of ALKBH5 is determined by target transcript identity, cellular context, reader environment, and upstream regulatory signals. This framework positions ALKBH5 as both a mechanistic hub and a context-guided therapeutic target.

## 1 Introduction

*N^6^*-methyladenosine (m^6^A) is the most abundant internal modification in eukaryotic mRNA and is a dynamic regulator of RNA metabolism, influencing transcript stability, splicing, nuclear export and translation (1, 2). The deposition, removal and interpretation of m^6^A marks are mediated by methyltransferases (“writers”), demethylases (“erasers”) and m^6^A-binding proteins (“readers”), respectively (3–6). Among the known m^6^A erasers, ALKBH5 has attracted increasing attention because of its broad involvement in cancer, immune regulation, cellular stress adaptation and viral infection (7–11).

**Figure 1:**
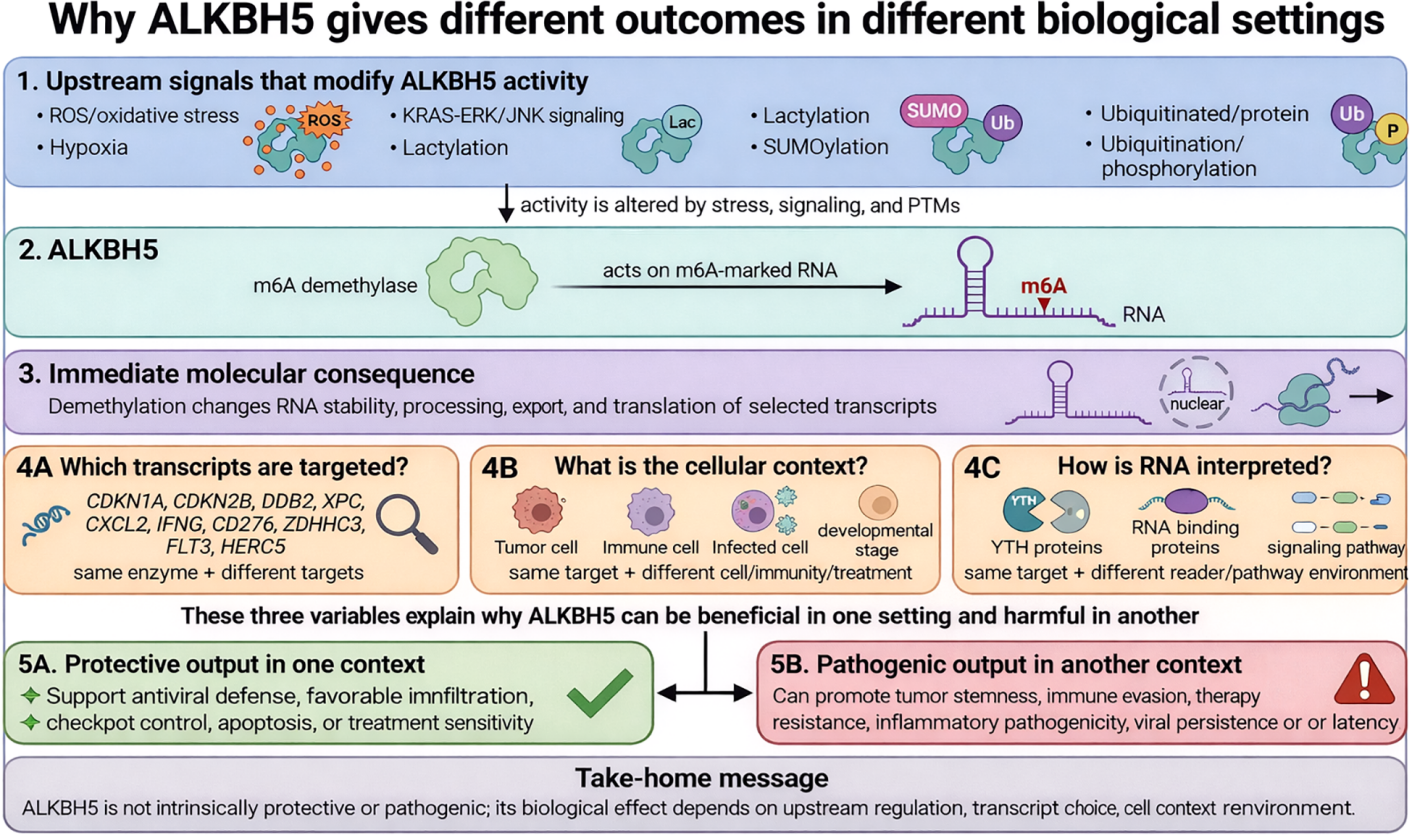
Mechanistic framework for context-dependent ALKBH5 function. ALKBH5 integrates upstream regulatory signals, including oxidative stress, hypoxia, oncogenic KRAS–ERK/JNK signaling, and post-translational modifications such as lactylation, SUMOylation, and ubiquitination/phosphorylation. Its core function is the demethylation of defined m^6^A-modified transcript classes, thereby altering RNA stability, processing, export, and translation. The downstream biological outcome is determined by transcript identity, reader context, cell type, and tissue state. Through these context-shaping variables, ALKBH5 can influence four major output modules: DNA damage response/checkpoint control, immune-cell differentiation and effector function, antiviral signaling/viral latency, and tumor stemness/therapy response. This framework emphasizes that ALKBH5 does not exert a single fixed biological effect; rather, its function becomes protective or pathogenic depending on upstream regulation, RNA target choice, and the cellular environment.

ALKBH5 is an m^6^A RNA demethylase that removes *N^6^*-methyladenosine marks from RNA and thereby regulates post-transcriptional RNA metabolism, including RNA stability, translation, and overall gene expression (7, 10). As one of the major erasers of this abundant RNA modification, ALKBH5 has attracted increasing attention because of its broad biological relevance (**Figure 1**). ALKBH5 helps maintain RNA metabolic balance, supports germ cell development and fertility, and influences cancer behavior, with changes in its expression associated with disease progression, prognosis, and response to therapy (8, 9, 11–14).

ALKBH5 exerts context-dependent effects in tumorigenesis and tumor progression by regulating the mRNA metabolism of both oncogenes and tumor suppressors. Increased ALKBH5 expression has been associated with aggressiveness in multiple cancers, including non-small cell lung cancer, glioblastoma, hepatocellular carcinoma, colorectal cancer, gastric cancer, endometrial cancer, and breast cancer (14–20). In contrast, ALKBH5 has also been reported to act as a tumor suppressor in certain settings, including colorectal cancer and hepatocellular carcinoma (21, 22). Nevertheless, its precise functions and underlying mechanisms in tumor development remain unclear and warrant further investigation.

T cells are central to adaptive immunity. They develop in the thymus into CD4^+^ and CD8^+^ subsets and, upon antigen encounter, differentiate into effector and memory populations that mediate immune regulation, cytotoxic defense, and long-term protection. During infection and tumor responses, T cells adopt diverse differentiation states shaped by transcriptional and epigenetic programs, whereas disruption of these regulatory pathways can contribute to autoimmune disease (23). m^6^A modification plays an important role in shaping T-cell development, differentiation, and function, and may therefore influence the overall behavior of adaptive immunity (24, 25). There is a pressing need to investigate the m^6^A demethylase ALKBH5 in T cell biology, given its central role in adaptive immunity and its promise as a therapeutic target.

A major limitation in the current literature is that ALKBH5 is often discussed separately within different biological domains, such as cancer, immunity or infection, without a unifying framework that explain why its effects differ so markedly across systems. As a result, the field remains fragmented and ALKBH5 is variably described as oncogenic, tumor suppressive, immunoregulatory, antiviral or proviral depending on the model examined (18, 21, 25, 26). Yet these labels are often based on downstream phenotypes rather than on mechanistic understanding of how ALKBH5 selects and regulates it direct RNA substrates.

In this review, we propose that diverse functions of ALKBH5 can be better understood through a context-dependent mechanistic framework. Under this view, the biological output of ALKBH5 is determined by the interaction of at least four major variables: (i) the identity of target transcripts and relevant m^6^A sites, (ii) the cellular context in which ALKBH5 acts, (iii) the RNA-binding and signaling environment that interprets the demethylated transcripts, and (iv) upstream regulatory inputs, including stress signals and post-transcriptional modifications of ALKBH5 itself (**Figure 1**). In this model, ALKBH5 is neither intrinsically protective nor intrinsically pathogenic; rather, its effect depends on which transcript programs dominate in a given biological setting.

Using this framework, we critically synthesize evidence on ALKBH5 in cellular stress adaptation and genome maintenance, immune-cell fate and function, host-pathogen interactions, and therapeutic targeting. We particularly emphasize convergent mechanisms, contradictory findings and unresolved questions that remain obscured when the literature is treated as a collection of isolated observations. By shifting the focus from descriptive cataloging to mechanistic integration, this review aims to clarify when and why ALKBH5 promotes beneficial adaptation, pathogenic signaling or therapeutic vulnerability.

## 2 The structure and function of ALKBH5

**Figure 2:**
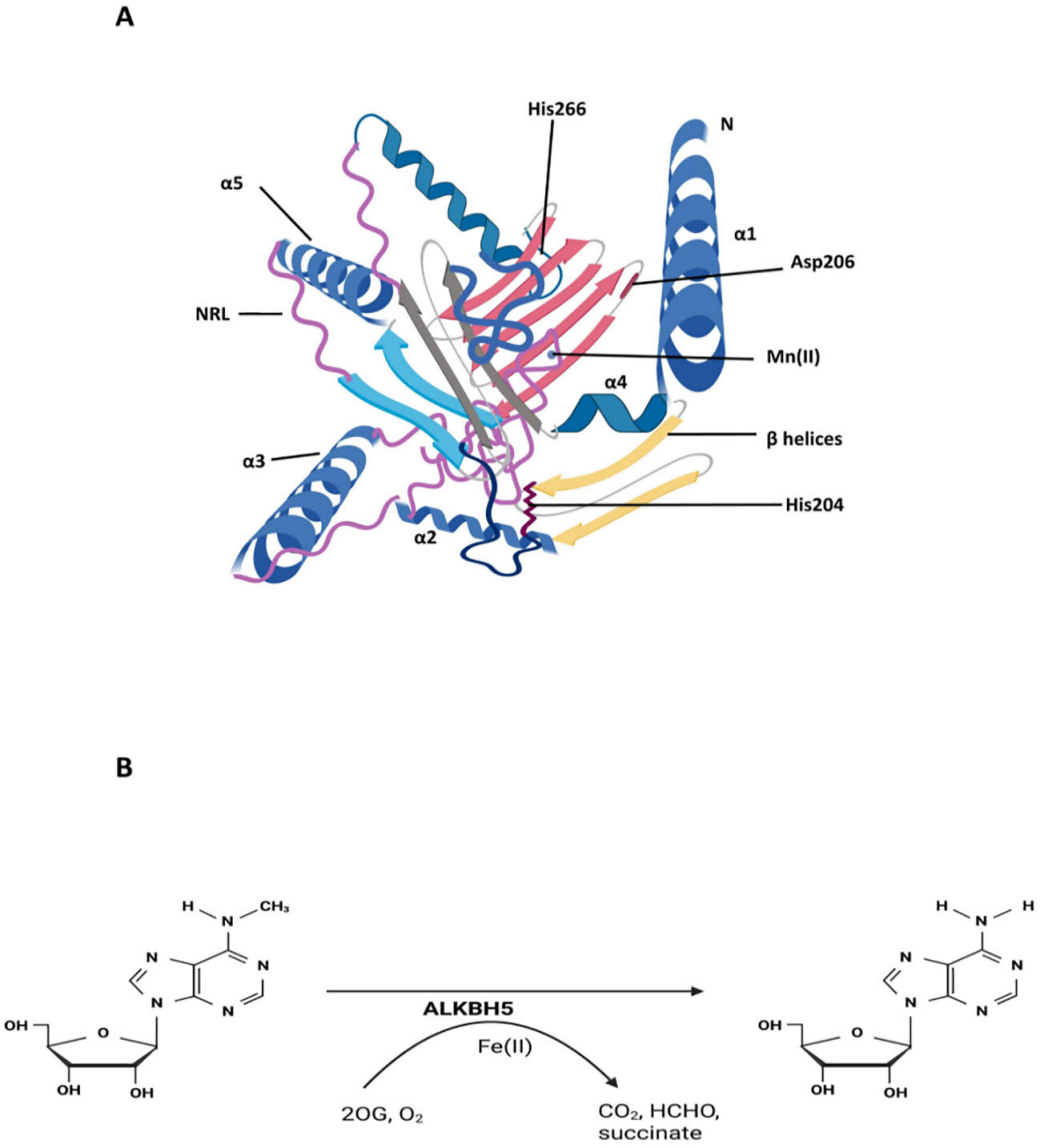
Structural features and catalytic mechanism of ALKBH5. **(A)** The structure of ALKBH5 is shown with the catalytic center of ALKBH5 which contains a double-stranded β-helix (DSBH) domain, composed of β-strands, α-helices, and nucleotide recognition loops (NRL). ALKBH5 bound to Mn²^+^, which substitutes for the catalytically active Fe²^+^, showing the active-site architecture within the double-stranded β-helix (DSBH) domain (32). The figure highlights the overall architecture of ALKBH5 and the organization of its catalytic core. **(B)** Catalytic mechanism of ALKBH5-mediated m^6^A demethylation. ALKBH5, an Fe(II)/2OG-dependent dioxygenase, oxidizes the *N^6^*-methyl group of adenosines to an unstable intermediate that decomposes to adenosine, releasing formaldehyde, succinate, and CO_2_. Figure concept adapted from (32).

ALKBH5 is a member of the iron (II)- and 2-oxoglutarate (2OG)-dependent AlkB oxygenase subfamily, the first group of 2OG oxygenases identified as nucleic acid N-demethylases (10, 27). This subfamily consists of nine members, including ALKBH1–8 and the fat mass and obesity-associated protein (FTO) (27). Endogenous ALKBH5 is predominantly localized in nuclear speckles, where it contributes to mRNA processing. As an mRNA-binding protein, its primary substrates are newly synthesized RNA transcripts (28–30). The ALKBH5 gene is located on chromosome 17p11.2 and encodes a 395-amino acid protein with a molecular weight of approximately 43 kDa. This protein catalyzes the oxidation of a broad range of substrates, including nucleic acids, lipids, proteins, and small-molecule metabolites (31). Structurally, the catalytic center of ALKBH5 contains a double-stranded β-helix (DSBH) domain, composed of 11 β-strands, 5 α-helices, and two nucleotide recognition loops (NRL1 and NRL2) (**Figure 2A**) (27). This DSBH domain is essential for its demethylase activity and indirectly modulates other functional domains involved in the demethylation process (33). The DSBH domain of ALKBH5 coordinates Fe²^+^ and 2OG, both of which are essential for its catalytic activity. Binding of Fe²^+^ and 2OG precedes the association of primary substrates—m^6^A-modified single-stranded nucleic acids—with the active site. Structural studies have shown that ALKBH5 exhibits greater conformational disorder in solution compared to its crystal structure, likely due to the absence of the Cys230–Cys267 disulfide bond, which limits 2OG binding to the catalytic pocket. Upon 2OG association, the enzyme undergoes a conformational rearrangement that enlarges the active site, enhances substrate affinity, and facilitates the entry of small-molecule substrates (34).

In the active site, a catalytically inert Mn²^+^ ion (substituted for the catalytically active Fe²^+^) is coordinated by the highly conserved metal-binding triad (His204, Asp206, and His266), together with water molecules and cofactors such as 2OG, N-oxalylglycine (NOG), or sulfate ions (**Figure 2A**). The structural configuration and coordination within this active site are critical for complex formation and catalytic function (31). Studies have also demonstrated the influence of Fe²^+^ on ALKBH5 function: coordination of Fe²^+^ within the enzyme forms an octahedral structure that enhances its stability and increases the hydrophobicity of residues surrounding the key HX(D/E) motif in the catalytic pocket (30). This hydrophobic environment facilitates substrate binding and catalysis by ALKBH5. Moreover, Fe²^+^ is essential for ALKBH5 interaction with both primary and secondary substrates (35). Notably, recent studies have also implicated ALKBH5 in the regulation of ferroptosis (36).

The demethylation mechanism of Fe(II)- and 2OG-dependent oxygenases proceeds through two main steps: dioxygen activation and substrate oxidation. In this process, Fe(II) and 2OG each provide two electrons to activate dioxygen, leading to the formation of a bridging peroxide and subsequently an Fe(IV)-oxo intermediate. This highly reactive species hydroxylates inert C–H bonds in RNA or other substrates, generating hydroxyl groups, releasing formaldehyde, and regenerating Fe(II) while converting 2OG to succinate. In the case of N-methylated substrates, hydrolytic deformylation enables direct demethylation (**Figure 2B**) (37, 38). ALKBH5 demethylates m^6^A in mRNA by oxidizing the *N^6^*-methyl group to an unstable hm^6^A intermediate (31), which spontaneously decomposes to adenosine within hours (39), releasing formaldehyde, succinate, and CO_2_ (38). As a result, only demethylated adenosine is detected following ALKBH5-mediated catalysis (40).

## 3 Mechanistic determinants of context-dependent ALKBH5 function

ALKBH5 is best understood as a context-dependent m^6^A demethylase: it removes m^6^A from selected RNAs and thereby changes RNA metabolism, including export, processing and overall fate, which is why it can influence many pathways rather than one fixed program (28). Its core catalytic effect is to reshape post-transcriptional regulation, so the same enzyme can alter stability, splicing, translation or retention of different transcripts depending on the target RNA and cell type (**Figure 1**) (28, 41). The specificity of ALKBH5 output depends first on which transcript and which m^6^A site are being demethylated, because different targets drive different biological consequences; for example, ALKBH5 can suppress pancreatic cancer through PER1 in a YTHDF2-dependent manner, showing that substrate choice is decisive (42). A second determinant is the reader context, as ALKBH5 does not function in isolation; by removing m^6^A, it alters the engagement of reader proteins and RNA-processing machinery with the transcript, thereby shifting its fate toward export, decay, stabilization, or modified translation (43). A third determinant is post-translational regulation of ALKBH5 itself, since ROS-induced SUMOylation can inhibit its demethylase activity during DNA-damage stress, whereas lactylation can enhance antiviral innate immunity against DNA herpesviruses and mpox virus, showing that ALKBH5 activity cannot be inferred from expression level alone (8, 44). A fourth determinant is subcellular localization, because nuclear retention of ALKBH5 changes which RNAs it can access and how those RNAs are processed as shown in EGFR-driven nuclear retention of ALKBH5 in glioblastoma (36). Finally, cell-state variables such as hypoxia, oxidative stress, oncogenic signaling, immune activation, and infection explain why ALKBH5 can appear beneficial in one setting but harmful in another: hypoxia-driven ALKBH5 can promote cancer stemness, ROS-linked ALKBH5 regulation can shape DNA-damage responses, and ALKBH5 can either suppress antiviral immunity through DDX46-mediated nuclear trapping of antiviral transcripts or enhance host defense in other viral settings, so the key question is not whether ALKBH5 is inherently protective or pathogenic, but what molecular context determines its output (**Figure 1**) (8, 44, 45).

## 4 ALKBH5 in cellular stress adaptation, genome maintenance and cell fate control

The m^6^A demethylase ALKBH5 has emerged as an important regulator of cellular stress adaptation, linking RNA methylation dynamics to genome maintenance, checkpoint control, and apoptotic fate decisions. Rather than functioning as a classical DNA repair factor, ALKBH5 appears to influence how cells respond to genotoxic stress by reshaping the stability, translation, and processing of transcripts involved in DNA damage signaling, cell cycle progression, and survival. ALKBH5 can either promote repair and recovery or facilitate pathological survival across DNA damage models, depending on the cellular context, the nature of the insult and the identity of the dominant RNA targets. This context-dependence is especially evident across studies of DNA damage response, cell cycle regulation and apoptosis.

### 4.1 ALKBH5 as a cellular stress-responsive regulator of the DNA damage response

ALKBH5 appears to function as a stress-responsive RNA regulator that links upstream damage signals to post-transcriptional control of genome maintenance. Under oxidative stress, reactive oxygen species induce SUMOylation of ALKBH5, which suppresses its demethylase activity and enables rapid transcriptional and post-transcriptional reprogramming of genes involved in diverse stress-adaptive processes, including DNA repair (8). This finding is important because it places ALKBH5 within a broader cellular stress network rather than depicting it as an isolated m^6^A eraser.

Within DNA damage response, ALKBH5 has been implicated in the regulation of multiple transcript classes with direct consequences for genome stability. In X-ray induced DNA damage models, ALKBH5 was shown to demethylate the transcripts of cyclin-dependent kinase inhibitors, including CDKN1A and CDKN2B, thereby reducing their stability and expression. Through this mechanism, ALKBH5 facilitates cell cycle progression and influences the cellular response to irradiation induced DNA damage (46). In hepatocytes exposed to radiation, ALKBH5 has also been reported to stabilize HMGB1 mRNA through m^6^A removal, thereby activating STING signaling and promoting apoptosis (47). Together, these studies suggest that ALKBH5 can affect both checkpoint signaling and downstream damage-associated pathways, although the phenotypic outcome depends on the biological setting.

Beyond checkpoint-associated transcripts, ALKBH5 influences genome maintenance through multiple mechanistic routes that vary across injury models and tumor contexts. ALKBH5 has been reported to participate in the repair of alkylation-associated damage and to mitigate cytotoxicity induced by alkylating agents (48). In gastric cancer, ALKBH5-mediated regulation of CHAC1 has been linked to malignant progression through effects on double-stranded DNA break-associated processes (49). In human bronchial epithelial cells, suppression of ALKBH5 contributed to Benzo(a)pyrene-induced upregulation of circ_0003552 and increased DNA damage burden (50). These observations support a broader role for ALKBH5 in stress-associated genome instability, although in several cases the precise direct RNA substrates responsible for the phenotype remain incompletely resolved.

### 4.2 ALKBH5 links DNA damage signaling to cell-cycle progression

Genome maintenance and cell-cycle control appear to be coordinated through ALKBH5-dependent regulation of RNA fate. This is mechanistically plausible, as successful adaptation to DNA damage requires tight coordination between checkpoint activation, repair, arrest and re-entry into proliferation. In some settings, ALKBH5 appears to weaken checkpoint restraint by destabilizing inhibitors such as CDKN1A and CDKN2B, thereby facilitating cell cycle progression after genotoxic stress (46). In other contexts, ALKBH5 promotes proliferative programs through the regulation of oncogenic or cell cycle associated transcripts. For example, in ovarian cancer, ALKBH5 was shown to accelerate cell cycle entry and epithelial-mesenchymal transition by activating CEP55 transcription through demethylation of FOXP2 mRNA (51). In endometrial cancer, ALKBH5-mediated m^6^A regulation of circ-NAB1 promoted EMT and cell cycle progression through the miR-876-3p/CDKN3 axis (19). These findings support a pro-proliferative role for ALKBH5 in several malignancies.

However, this effect is not universal. In multiple myeloma, depletion of ALKBH5 increased the proportion of cells in G0/G1 phase, reduced the G2/M population, decreased DNA synthesis and promoted apoptosis, thereby suppressing tumor progression (52). Similar anti-proliferative effects of ALKBH5 inhibition have been observed in glioblastoma stem-like cells, where ALKBH5 loss increased the G0/G1 arrest and reduced S and G2/M phase populations (53), and in non-small cell lung cancer, where ALKBH5 knockdown induced G1 arrest and apoptosis (54). Thus, the available data do not support a simple classification of ALKBH5 as a uniformly pro or anti-cell cycle. Rather, ALKBH5 appears to regulate the balance between checkpoint enforcement and proliferative recovery in a transcript and context-dependent manner.

### 4.3 Context-dependent effects of ALKBH5 on apoptosis

Apoptotic regulation provides another example of the context dependence of ALKBH5 function. In several tumor models ALKBH5 supports survival and treatment resistance by sustaining stemness-associated or growth-promoting programs. In glioma, the lncRNA SOX2OT recruits ALKBH5 to demethylate SOX2 transcripts, thereby enhancing SOX2 expression, suppressing apoptosis, promoting proliferation and conferring temozolomide resistance (55). In acute leukemia, ALKBH5 knockdown significantly increased apoptosis in both leukemia cells and leukemia stem cells, indicating that ALKBH5 can function as a pro-survival factor in this context (56, 57). Long-term cadmium exposure has also been associated with reduced ALKBH5 expression and altered m^6^A patterns, contributing to a malignant phenotype characterized by enhanced proliferation, invasion and apoptosis resistance (58).

By contrast, several studies indicate that ALKBH5 can also promote apoptotic responses under selected conditions. In osteosarcoma, ALKBH5 overexpression reduced m^6^A methylation of pre-miR-181b-1 and YAP mRNA and significantly induced apoptosis (59). In another osteosarcoma model, elevated ALKBH5 expression decreased SOCS3 mRNA stability in an m^6^A-dependent manner, thereby inactivating STAT3 signaling and promoting apoptosis (60). Conversely, ALKBH5-mediated m^6^A deficiency has also been reported to upregulate USP22 and RNF40, promoting osteosarcoma growth and proliferation (61). These divergent findings underscore that ALKBH5 does not exert a fixed apoptotic role; instead, apoptosis appears to reflect the cumulative effect of ALKBH5 on broader transcript networks governing stress tolerance, survival signaling and malignant fitness.

Overall, current evidence supports a model in which ALKBH5 couples cellular stress cues to post-transcriptional programs that influence DNA repair, checkpoint control and apoptotic fate. Yet the literature does not support a single unified role for ALKBH5 in these processes. These discrepancies likely arise from differences in cell type, damage source, oncogenic background, post-translational regulation of ALKBH5 and the identity of dominant downstream RNA targets.

## 5 ALKBH5 in immune-cell fate, pathogenicity, and tumor-immune crosstalk

Although genome maintenance and immune regulation are often discussed as separate biological themes, both require rapid transcriptome remodeling under conditions of stress and activation. In this sense, the role of ALKBH5 in DNA damage response, checkpoint control and survival may reflect a broader function in coordinating cellular adaptation through selective RNA demethylation. This framework also provides a mechanistic bridge to immune cell biology, where activated lymphocytes must similarly balance proliferation, differentiation and persistence. Compared with well-established roles of m^6^A writers in lymphocyte biology, the contribution of the demethylase ALKBH5 to immune regulation remains less comprehensively defined. In this review, we have focused on the roles of ALKBH5 in T cell biology.

### 5.1 ALKBH5 in early T-cell development decisions

A central issue in immune development is whether ALKBH5 acts at specific lineage checkpoints rather than across all stages of T-cell maturation. ALKBH5 has been shown to play a crucial role in the early development of murine γδ T cells, acting as a potential checkpoint in the fate decision between αβ and γδ T cell lineages (62). Mechanistically, ALKBH5 modulates the expression of Jagged1 and Notch2 in lymphocytes, thereby altering the Jagged1/Notch2 signaling, impairing γδ T cell precursor development and ultimately reducing the mature γδ T cell (62). This finding is important because it places ALKBH5 at the level of lineage commitment rather than limiting its role to mature cell function.

However, the developmental role of ALKBH5 is not uniformly supported across all T-cell contexts. Under steady-state conditions, T-cell development at later stages in the thymus and in peripheral lymphoid tissues was reported to remain largely unaffected in CD4^+^ T cell-specific ALKBH5-deficient mice (26). Likewise, METTL3 and METTL14, but not ALKBH5, were reported to contribute to T follicular helper (Tfh) cell development (63). These observations suggest that ALKBH5 may not function as a universal regulator of all T-cell developmental programs. Instead, its developmental effects may be restricted to specific checkpoints, lineages or activation states. This distinction is important for a critical reading of the literature: rather than concluding broadly that ALKBH5 controls “T-cell development,” the current data support a more nuanced view in which ALKBH5 has selective roles in particular developmental trajectories, especially those involving γδ T-cell differentiation.

### 5.2 ALKBH5 in CD4^+^ T-cell activation and pathogenicity

Beyond developmental effects, ALKBH5 has also been implicated in the pathogenic programming of activated CD4^+^ T cells. ALKBH5 mRNA expression was found to be upregulated in Th1, Th2, Th17, and Treg cells, while FTO expression remained largely unchanged compared with naïve CD4^+^ T cells (26). This differential expression pattern suggests that ALKBH5 is dynamically engaged during CD4^+^ T-cell activation and differentiation, although expression alone does not establish functional necessity.

The clearest functional evidence comes from inflammatory disease models, in which CD4^+^ T cell-specific deletion of ALKBH5 rendered mice resistant to autoimmune colitis and experimental autoimmune encephalomyelitis (EAE), accompanied by reduced neutrophil recruitment into the central nervous system (26). Mechanistically, ALKBH5 deficiency decreased the stability of CXCL2 and IFN-γ transcripts by promoting RNA decay, thereby limiting CD4^+^ T-cell pathogenicity (**Table 1**) (26). These findings support a model in which ALKBH5 enhances inflammatory T-cell programs by stabilizing key effector-associated transcripts.

**Table 1:** Summary Table of ALKBH5 roles in T cell biology.

T cell type/Context	Role of ALKBH5	Mechanism/findings	References
γδ T cells	Regulates early development, acts as checkpoint between αβ and γδ T cells	Alters Jagged1/Notch2 signaling, reduces mature γδ T cells	(62)
General T cell development (CD4^+^ T cell-specific KO mice)	Dispensable in later thymic and peripheral development	No significant defects in steady state development	(26)
CD4^+^ T cell (autoimmunity models)	Promotes pathogenicity; ALKBH5 deletion confers resistance	KO mice resistant to colitis & EAE; reduced neutrophil CNS infiltration due to decreased stability of CXCL2 & IFN-γ mRNA	(26)
CD4^+^ T cell subsets (Th1, Th2, Th17, Treg)	ALKBH5 expression increases upon differentiation	FTO expression unchanged; ALKBH5 regulates RNA stability	(26)
CD8^+^ T cells (colorectal cancer)	Supports CD8^+^ infiltration; enhances anti-tumor activity	ALKBH5 expression linked to better prognosis and reduced CRC proliferation	(21)
CD8^+^ T cells (cytotoxicity regulation)	Loss of ALKBH5 impairs cytotoxicity	Lower ALKBH5→stabilized CD276 mRNA→CRC immune evasion by suppressing cytotoxic CD8^+^ T cells	(64)
Mature T cells (lupus erythematosus)	Promotes apoptosis; inhibits proliferation	ALKBH5 overexpression increases apoptosis of mature T cells	(65)
Inflammatory regulation (neutrophils & macrophages)	Influences inflammatory cell function	CD4^+^ T cells produce IFN-γ → macrophage activation; ALKBH5 impacts this axis	(26)
PD-1/PD-L1 immune checkpoint (glioma)	Enhances anti-tumor immunity	ALKBH5 reduces PD-L1 via ZDHHC3 mRNA	(66)

At the same time, these data also illustrate an important point about context. The same demethylase that appears dispensable for some steady-state developmental processes can become functionally important during inflammatory activation. Thus, ALKBH5 may be less relevant for baseline T-cell maintenance than for reinforcing disease-driving transcriptional and post-transcriptional programs under pathogenic conditions. This distinction is explicit, because it helps explain why some studies report limited developmental phenotypes while others observe strong effects in inflammatory disease models.

### 5.3 ALKBH5 in CD8^+^ T-cell responses and anti-tumor immunity

ALKBH5 also plays an important role in tumor immunity, particularly by influencing CD8^+^ T-cell infiltration and cytotoxic activity. In colorectal cancer, higher ALKBH5 expression has been associated with increased CD8^+^ T-cell infiltration at tumor sites, reduced tumor cell proliferation and improved patient prognosis (**Table 1**) (21). This observation suggests that in some tumor settings, ALKBH5 expression correlates with a more favorable immune microenvironment.

However, this is not uniform tumor-associated immune effect reported for ALKBH5. In colorectal cancer, reduced ALKBH5 expression was shown to enhance the stability and expression of CD276 mRNA, thereby promoting immune evasion through suppression of cytotoxic T-cell function (64). This finding provides a plausible mechanistic explanation for how ALKBH5 loss may impair anti-tumor immunity in at least some colorectal tumors. Taken together, these studies indicate that ALKBH5 can influence not only the abundance of infiltrating CD8^+^ T-cells but also the functional state of tumor-immune interactions through regulation of immune checkpoint-associated molecules.

ALKBH5 contributes to anti-tumor immune regulation by suppressing PD-L1 expression through modulation of ZDHHC3 mRNA stability in glioma. (66). This reduction in PD-L1 was associated with enhanced infiltration of cytotoxic lymphocytes and increased levels of proinflammatory cytokines in the cerebrospinal fluid (66). In this context, ALKBH5 appears to counteract immune suppression and favor anti-tumor immune activity. These observations are valuable because they show that ALKBH5 is not simply a tumor-intrinsic growth regulator. Rather, it can reshape tumor-immune crosstalk by altering checkpoint-related or immunoregulatory transcripts. At the same time, the direction of this effect depends heavily on tumor type, transcript target and local microenvironment. Therefore, it would be inaccurate to characterize ALKBH5 as either uniformly immunostimulatory or uniformly immunosuppressive in cancer. A more defensible conclusion is that ALKBH5 modulates antitumor immunity in a context-dependent manner through effects on both immune-cell-infiltration and tumor immune evasion programs.

### 5.4 ALKBH5 in broader immune-cell crosstalk and inflammatory microenvironments

ALKBH5-dependent immune regulation also extends beyond T-cell-intrinsic programs into broader inflammatory and multicellular signaling networks. The available literature points to broader roles in inflammatory cell crosstalk. CD4^+^ T lymphocytes, for example, secrete IFN-γ, which in turn activates macrophages (67), highlighting how ALKBH5-dependent regulation of T-cell cytokine output may secondarily influence innate immune compartments. This broader perspective is useful because it expands ALKBH5 from being merely a regulator of isolated T-cell subsets to a modifier of multicellular immune networks. This broader immunological role is also evident in non-tumor inflammatory settings. In a male model of kidney injury, inhibition of ALKBH5 enhanced m^6^A modification and increased stability of CCL28 mRNA, thereby promoting Treg recruitment and reducing macrophage and neutrophil infiltration (68). This finding suggests that ALKBH5 can also influence tissue inflammation indirectly by modulating chemokine expression and immune cell recruitment patterns. Likewise, in a case-control study of lupus erythematosus, ALKBH5 overexpression promoted apoptosis of mature T-cells while inhibiting their proliferation (65), indicating that ALKBH5 may also affect peripheral T-cell homeostasis under autoimmune conditions.

These findings broaden the immunological significance of ALKBH5 but also complicate interpretation. Not all observed immune phenotypes are necessarily T-cell intrinsic, and not all are likely to be governed by the same molecular logic. Some reflect transcript stabilization in activated T-cells, whereas others involve chemokine-mediated recruitment, immune checkpoint regulation or changes in mature T cell survival. This heterogeneity should be acknowledged directly rather than smoothed over, because it is central to understanding why the ALKBH5 literature appears fragmented. Thus, the shared principle is not that ALKBH5 always enhances or suppresses immunity. Rather, ALKBH5 tunes immune outcomes by altering the post-transcriptional fate of immune relevant transcripts in a manner that depends on cell identity, activation state, tissue context and disease environment. This framework is more consistent with the available evidence than any single direction model and better accommodates the apparently divergent observations across the development, inflammation, autoimmunity and cancer.

## 6 Implications of ALKBH5 in viral diseases and therapeutics

The immunoregulatory roles of ALKBH5 also have direct implications for host-pathogen interactions. Viral infection places intense pressure on the host to rapidly reprogram innate immune signaling, metabolism, and RNA processing, while many viruses exploit the same post-transcriptional pathways to promote persistence or immune evasion. The disease relevance of ALKBH5 extends beyond cancer and immune cell biology to host-pathogen interactions, where m^6^A dependent control of RNA fate can influence anti-viral signaling, viral persistence, latency, and virus-associated oncogenesis. However, the available evidence does not support a single uniform role for ALKBH5 in infection. In some settings, ALKBH5 enhances host defense by sustaining antiviral gene expression or interferon associated pathways, whereas in other it suppresses innate immunity by demethylating immune transcripts in ways that limit their translation or retain them in the nucleus. This apparent contradiction is better understood as a sequence of transcript- and context specific regulation rather than as a single inconsistency. Viewed in this way, ALKBH5 emerges as a context-dependent regulator of host-pathogen conflict whose therapeutic value depends on its whether dominant effect is antiviral, proviral, latency-supportive or transformation promoting.

### 6.1 ALKBH5 in innate antiviral signaling and host defense

ALKBH5 contributes to innate host defense, but its antiviral effects vary depending on the pathogen and the regulated transcript network (69, 70). It has been shown to regulate IFN-β production following human cytomegalovirus (HCMV) infection of dsDNA stimulation, supporting a role in antiviral host-defense (71). In addition to its direct demethylase activity, ALKBH5 can also be regulated by post-translational modifications, thereby adding another layer of context dependence to its immune function. For example, lactylation of ALKBH5 was reported to enhance immune defense against DNA herpesviruses and mpox virus, suggesting that ALKBH5 activity may be dynamically tuned by infection-associated metabolic and signaling changes (44).

ALKBH5 has also been implicated in response to RNA viruses, although the direction of its effect remains debated. One study showed that DDX46 recruits ALKBH5 to demethylate MAVS, TRAF3 and TRAF6 transcripts, thereby suppressing type I interferon production (**Table 2**) (45). Consistent with this model, ALKBH5 knockdown significantly enhanced type I interferon responses during VSV infection, indicating that ALKBH5 can function as a negative regulator of innate immunity in this context (45). However, an *in-vivo* study reported reduced serum IFN-β levels in *Alkbh5*-deficient mice after VSV infection, suggesting instead that ALKBH5 may act as a positive regulator of IFN-β production (72). These seemingly opposing findings highlight an important point: ALKBH5 does not appear to exert a fixed antiviral function, and its biological effect depends on the identity of the regulated transcript set and the infection model under investigation.

**Table 2:** Summary Table of ALKBH5 roles in viral diseases.

Disease	ALKBH5 Up/Down	Target	Function	References
HCMV/dsDNA infection	Up	IFN-β	Regulates IFN-β Production during infection	(71)
DNA herpesviruses/mpox virus	Up	Innate immunity pathways	Lactylation of ALKBH5 promotes innate immunity	(44)
VSV infection	Up	MAVS, TRAF3, TRAF6	Removes m^6^A to suppress type I IFNs; knockdown increases IFNs	(45)
VSV infection	Down	IFN-β	Loss of ALKBH5 reduces serum IFN-β, suggesting positive regulation	(72)
Cervical cancer (HPV)	Up	PAK5	Promotes tumorigenesis and metastasis	(73)
General viral infection	Up	Antiviral transcripts	DDX46 recruits ALKBH5 to demethylate antiviral transcripts, retain in nucleus, inhibit IFN production	(45)
PEDV infection	Down	*IFIT3, HERC5*	Deletion downregulates antiviral genes, activates IRF3/TBK1, enhances *ISG15*-mediated responses	(74)
HIV-1 latency	Down	m^6^A-modified trancripts	Inhibition of ALKBH5 facilitates reactivation of latent virus (‘shock and kill’ strategy)	(75)
General viral infection/OGDH-itaconate pathway	Down	OGDH	Reduced ALKBH5 activity downregulates OGDH-itaconate pathway, inhibits viral replication	(72)

ALKBH5 has also been shown to support antiviral defense in IPEC-J2 cells infected with porcine epidemic diarrhea virus (PEDV). In this system, deletion of ALKBH5 downregulated the antiviral genes IFIT3 and HERC5, which activate the IRF3/TBK1 signaling pathway, while HERC5 further promotes antiviral responses through ISG15 (74). These findings suggest that ALKBH5 can sustain antiviral gene expression in certain epithelial infection settings. Taken together, the literature indicates that ALKBH5 can either reinforce or suppress antiviral immunity depending on whether its dominant targets promote interferon signaling, antiviral effector pathways, or inhibitory RNA retention programs.

### 6.2 ALKBH5 in viral persistence, latency and virus-associated pathogenesis

Beyond acute antiviral signaling, ALKBH5 also influences viral persistence and infection-associated disease states. In cervical cancer linked to human papillomavirus (HPV), the viral E6/E7 oncoproteins were shown to alter global m^6^A modification and upregulate ALKBH5 expression, thereby promoting tumorigenesis and metastasis through regulation of PAK5 (73). This observation is important because it shows ALKBH5 can contribute not only to host antiviral responses, but also to the pathogenic reprogramming of host cells in the setting of persistent viral oncogenesis.

HIV-1 latency represents a mechanistically distinct setting in which ALKBH5 influences viral persistence rather than acute antiviral restriction. Inhibition of ALKBH5 has been shown to facilitate reactivation of latent HIV-1, aligning with the “shock and kill” strategy aimed at exposing dormant virus immune or pharmacological clearance (**Table 2**) (75). This finding highlights the role of m^6^A RNA regulation in maintaining HIV latency and positions ALKBH5 as a potential target, reawakening latent virus. Mechanistically, this is especially relevant because it shifts the question from acute antiviral restriction to post-transcriptional control of viral persistence.

ALKBH5 also affects infection outcome through metabolic rewiring, broadening its role beyond canonical interferon signaling. During infection, host cells can alter metabolism through m^6^A-dependent mechanisms to resist viral replication in an interferon-dependent manner. Reduced enzymatic activity of ALKBH5 was shown to downregulate the OGDH-itaconate pathway through YTHDF2, thereby suppressing viral replication (72). This finding broadens the functional scope of ALKBH5 by showing that its disease relevance is not limited to canonical interferon pathways, but also includes metabolic control of infection outcomes.

### 6.3 Therapeutic implications of ALKBH5: inhibition, restoration and context-guided targeting

The broad involvement of ALKBH5 in viral infection, cancer progression, DNA damage responses and therapeutic responses makes it an attractive translational candidate. However, the same feature that makes ALKBH5 biologically important─ its context dependent function─ also complicates therapeutic development. The current literature suggests that both inhibition and restoration of ALKBH5 activity may be beneficial, depending on disease type, cellular-context and dominant downstream targets.

HIV latency reversal is currently one of the clearest contexts in which ALKBH5 inhibition may have therapeutic value. There has been evidence suggesting that inhibition of ALKBH5-mediated pathways enhances the HIV-1 reactivation from latency. Specifically, the ALKBH5 inhibitor 3 (ALKi-3) potentiated romidepsin-induced viral reactivation in *in-vitro* latency models, introducing the concept of “dual-kick” approach that targets both transcriptional and post-transcriptional mechanisms to promote HIV-1 reactivation (75). This represents a particularly clear example in which ALKBH5 inhibition may have translational value.

Cancer provides an additional context in which ALKBH5-directed intervention may be therapeutically relevant. In X-ray-induced DNA damaged models, ALKBH5 depletion attenuated DNA damage responses by arresting cell-cycle progression and promoting apoptosis, suggesting that ALKBH5 may represent a target in DNA damage-based cancer therapies (46). In gastric cancer, ALKBH5 act as an oncogenic factor by demethylating CHAC1 mRNA, reducing CHAC1 expression and promoting tumorigenesis and metastasis through the ALKBH5/CHAC1/ROS axis (49). These findings support the view that ALKBH5 may serve both as a prognostic marker and as a therapeutic target in selected malignancies.

In contrast, other disease settings suggest that restoring or preserving ALKBH5-dependent regulation may be more beneficial than direct inhibition. In KRAS- mutant NSCLC platinum resistance was driven by ERK/JNK-mediated inhibition of ALKBH5 demethylase activity through post-translational modification. Disrupting this KRAS-driven m^6^A dysregulation, either by overexpressing a SUMOylation deficient ALKBH5 mutant or by pharmacologically inhibiting METTL3, significantly resensitized KRAS-mutant NSCLC cells to platinum treatment *in vitro* and *in vivo* (76). This is an important example because it shows that therapeutic intervention may not always require direct inhibition of ALKBH5; in some cases, restoring ALKBH5-dependent regulation may be more effective.

Glioblastoma illustrates the same principle: therapeutic benefit may arise from correcting ALKBH5-associated pathway dysregulation rather than from uniformly inhibiting the enzyme. The MST4–USP14–ALKBH5 signaling axis has been shown to regulate stemness, homologous recombination-mediated repair of DNA double-strand breaks, radioresistance, and tumorigenicity in glioblastoma stem cells. Pharmacologically inhibition of USP14 with IU1 disrupted ALKBH5 deubiquitylation and improved the efficacy of irradiation therapy in xenograft models, highlighting this pathway as a promising therapeuting target in GBM (77). By contrast, cadmium exposure was reported to suppress ALKBH5, leading to elevated m^6^A methylation, dysregulated NF-kB and NRF2, oxidative stress, inhibition of apoptosis and malignant transformation. In this setting, targeting m^6^A-associated-pathways may help counteract cadmium-induced malignancy (58). Overall, the therapeutic significance of ALKBH5 lies in its position at a key regulatory node where RNA methylation can redirect disease-relevant cell states. Yet the current evidence does not justify a simplistic conclusion that ALKBH5 should always be inhibited or always be restored. Together, these studies reinforce the conclusion that therapeutic strategies must be aligned with the specific pathological context rather than based on a one-direction-fits-all-model.

## 7 Conclusion and future perspectives

**Figure 3:**
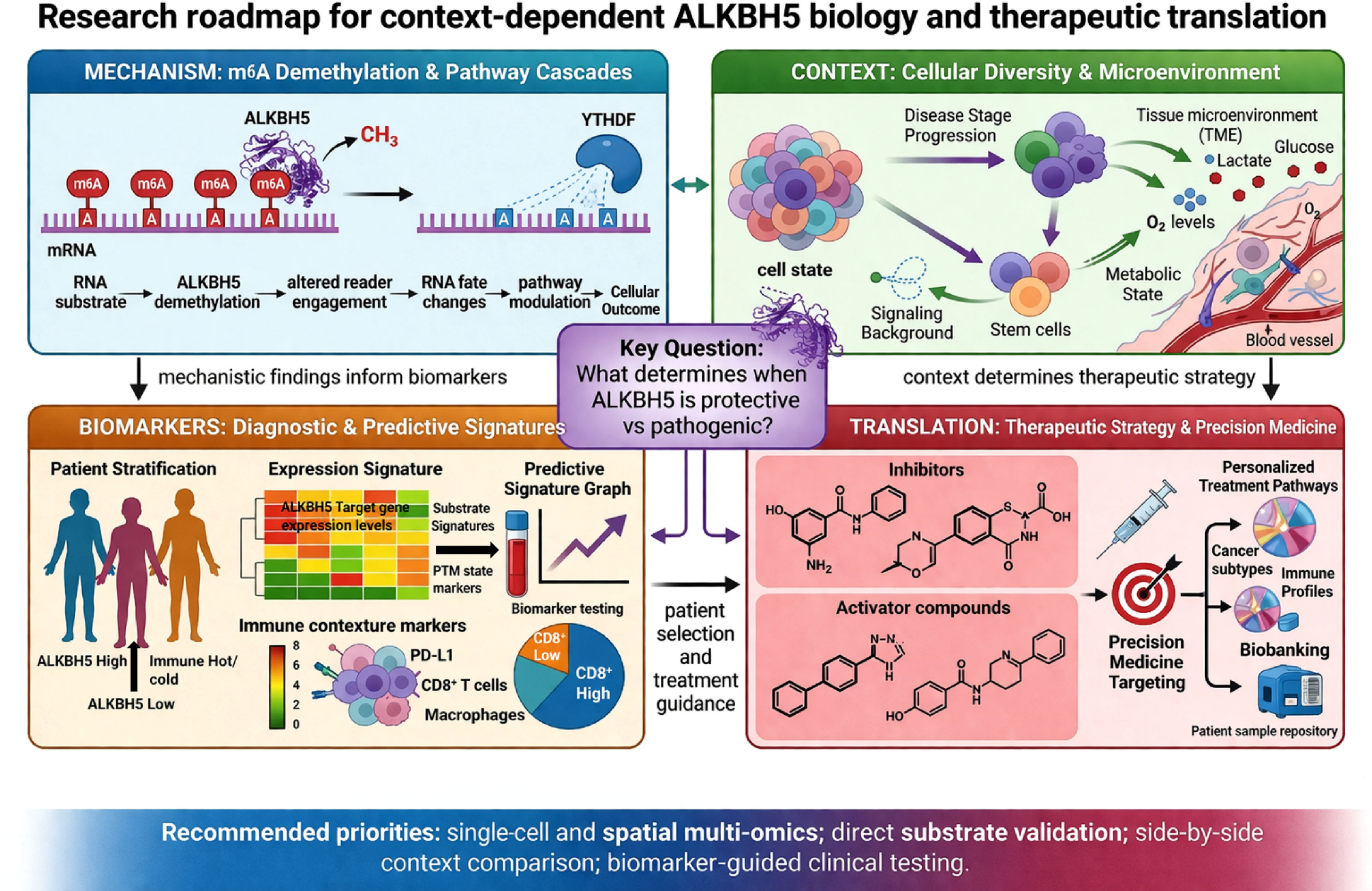
Research roadmap for context-dependent ALKBH5 biology and therapeutic translation. The central unresolved question in the field is what determines whether ALKBH5 exerts protective or pathogenic effects in a given biological context. Four interconnected research priorities are highlighted. Mechanism focuses on defining direct ALKBH5 substrates, m6A sites, reader dependencies, and RNA fate outcomes. Context addresses the influence of cell type, disease stage, tissue niche, post-translational modifications, metabolic state, and signaling background on ALKBH5 function. Biomarkers emphasize patient stratification using ALKBH5 expression, substrate signatures, post-translational state, and immune contexture. Translation focuses on context-matched therapeutic strategies, including inhibition, activation, or pathway correction. The connecting arrows indicate that mechanistic findings inform biomarker development, contextual variables determine therapeutic direction, and integrated analysis is required to explain why the same demethylase can produce divergent biological outcomes.

ALKBH5 has emerged as a major epitranscriptomic regulator of genome maintenance, immune-cell function, host-pathogen interactions, and therapeutic response. However, the field has moved beyond asking whether ALKBH5 is simply beneficial or harmful. The central unresolved question is what determines when ALKBH5 becomes protective in one context but pathogenic in another. Addressing this question will require a research strategy that moves from mechanism to translation in a defined order. First, future studies should identify the direct RNA substrates, m^6^A sites, and reader dependencies that mediate ALKBH5 function in specific cell types and disease settings. Second, these findings must be integrated with contextual variables, including cell lineage, disease stage, tissue niche, metabolic state, and post-translational modification of ALKBH5, to explain why the same demethylase produces divergent outcomes (**Figure 3**). Third, these mechanistic insights should be translated into biomarker frameworks that allow patient stratification on the basis of ALKBH5 expression, substrate signatures, and activity state. Only then can context-matched therapeutic strategies, including inhibition, restoration, or pathway-level correction, be rationally developed (**Figure 3**). Because most available evidence remains preclinical, future work should prioritize cell-type-specific *in-vivo* models, direct substrate validation, and biomarker-guided clinical studies to determine when ALKBH5 can be safely and effectively targeted. In this way, the therapeutic promise of ALKBH5 will depend not only on its therapeutic targetability, but on the precision with which its context-dependent biology is understood.
